# The Feasibility of Implementing Mainstream Germline Genetic Testing in Routine Cancer Care—A Systematic Review

**DOI:** 10.3390/cancers14041059

**Published:** 2022-02-19

**Authors:** Kyra Bokkers, Michiel Vlaming, Ellen G. Engelhardt, Ronald P. Zweemer, Inge M. van Oort, Lambertus A. L. M. Kiemeney, Eveline M. A. Bleiker, Margreet G. E. M. Ausems

**Affiliations:** 1 Division Laboratories, Pharmacy and Biomedical Genetics, Department of Genetics, University Medical Center Utrecht, Heidelberglaan 100, 3584 CX Utrecht, The Netherlands; k.bokkers@umcutrecht.nl (K.B.); m.vlaming@umcutrecht.nl (M.V.); 2 Division of Psychosocial Research and Epidemiology, The Netherlands Cancer Institute, Plesmanlaan 121, 1066 CX Amsterdam, The Netherlands; e.engelhardt@nki.nl (E.G.E.); e.bleiker@nki.nl (E.M.A.B.); 3 Department of Gynecological Oncology, University Medical Center Utrecht, Heidelberglaan 100, 3584 CX Utrecht, The Netherlands; r.zweemer@umcutrecht.nl; 4 Department of Urology, Radboud University Medical Center, Geert Grooteplein Zuid 10, 6525 GA Nijmegen, The Netherlands; inge.vanoort@radboudumc.nl (I.M.v.O.); bart.kiemeney@radboudumc.nl (L.A.L.M.K.); 5 Department for Health Evidence, Radboud University Medical Center, Geert Grooteplein Zuid 21, 6525 EZ Nijmegen, The Netherlands; 6 Department of Clinical Genetics, Leiden University Medical Center, Albinusdreef 2, 2333 ZA Leiden, The Netherlands; 7 Family Cancer Clinic, The Netherlands Cancer Institute, Plesmanlaan 121, 1066 CX Amsterdam, The Netherlands

**Keywords:** genetic counseling, mainstream genetic testing, cancer, feasibility, quality of care, systematic review

## Abstract

**Simple Summary:**

Germline genetic testing for patients with cancer can have important implications for treatment, preventive options, and for family members. In a mainstream genetic testing pathway, pre-test counseling is performed by non-genetic healthcare professionals, thereby making genetic testing more accessible to all patients who might benefit from it. These mainstream genetic testing pathways are being implemented in different hospitals around the world, and for different cancer types. It is important to evaluate how a mainstream genetic testing pathway can be made sustainable and if quality of genetic care is maintained. We show in this systematic review that it is feasible to incorporate a mainstream genetic testing pathway into routine cancer care while maintaining quality of care. A training procedure for non-genetic healthcare professionals and a close collaboration between genetics and other clinical departments are highly recommended to ensure sustainability.

**Abstract:**

Background: Non-genetic healthcare professionals can provide pre-test counseling and order germline genetic tests themselves, which is called mainstream genetic testing. In this systematic review, we determined whether mainstream genetic testing was feasible in daily practice while maintaining quality of genetic care. Methods: PubMed, Embase, CINAHL, and PsychINFO were searched for articles describing mainstream genetic testing initiatives in cancer care. Results: Seventeen articles, reporting on 15 studies, met the inclusion criteria. Non-genetic healthcare professionals concluded that mainstream genetic testing was possible within the timeframe of a routine consultation. In 14 studies, non-genetic healthcare professionals completed some form of training about genetics. When referral was coordinated by a genetics team, the majority of patients carrying a pathogenic variant were seen for post-test counseling by genetic healthcare professionals. The number of days between cancer diagnosis and test result disclosure was always lower in the mainstream genetic testing pathway than in the standard genetic testing pathway (e.g., pre-test counseling at genetics department). Conclusions: Mainstream genetic testing seems feasible in daily practice with no insurmountable barriers. A structured pathway with a training procedure is desirable, as well as a close collaboration between genetics and other clinical departments.

## 1. Introduction

The use of germline genetic tests in cancer care is changing rapidly. Gene panel testing is increasingly being used instead of single gene testing, and the criteria for genetic testing have been broadened in several types of cancer [1,2,3]. In addition, new treatment options are now available that depend on the results of genetic testing. For example, poly (ADP-ribose) polymerase (PARP) inhibitors can be used in the treatment of patients with ovarian, breast, or prostate cancer, and they are especially beneficial for patients carrying a germline or somatic pathogenic variant in a *BRCA* gene [4,5,6]. These changes in genetic testing and care, together with the growing numbers of eligible patients who can benefit from genetic testing and the limited capacity of the genetics departments to meet the needs of the increasing numbers of patients, have paved the way for mainstream genetic testing. In a mainstream genetic testing pathway, non-genetic healthcare professionals (NGHCPs) provide pre-test counseling (e.g., review cancer family history, discuss possible implications of a genetic test) and order the genetic test after obtaining informed consent. These NGHCPs are not formally trained as genetic counselors or clinical geneticists. In the standard genetic testing pathway, these steps are taken by genetic counselors or clinical geneticists.

The development and implementation of a mainstream genetic testing pathway was first described by George et al. in 2016 in ovarian cancer patients, within the Mainstreaming Cancer Genetics program [7]. Subsequently, several other mainstream genetic testing initiatives arose around the globe in a research setting, mainly in ovarian cancer, but also in other cancers [8,9,10,11]. 

The systematic review of Scheinberg et al. studied the acceptability of mainstream genetic testing [12]. They showed that mainstream genetic testing in cancer care was acceptable for patients and NGHCPs to manage the growing demand for genetic tests. They also concluded that mainstream genetic testing was feasible. To make recommendations for implementing a mainstream genetic testing pathway in daily practice, it is important to determine clear end points for feasibility and to guarantee that quality of care is maintained. To maintain quality of care, NGHCPs should be well equipped to perform pre-test counseling. The systematic review of Scheinberg et al. included studies on ovarian, breast and colorectal cancer [12]. Since then, multiple new mainstream initiatives in different types of cancers have been published. 

We performed a systematic review to assess the available literature on mainstream genetic testing in cancer care. The following research questions will be answered: (1) Is mainstream genetic testing in cancer patients feasible for NGHCPs in daily practice? and (2) Is the quality of genetic care maintained when patients undergo mainstream genetic testing?

## 2. Materials and Methods

This review was conducted according to the guidelines established by the Preferred Reporting Items for Systematic Reviews and Meta-Analyses (PRISMA) report [13].

### 2.1. Eligibility Criteria

Studies were eligible for inclusion if a mainstream genetic testing pathway in patients with cancer was evaluated. This pathway had to meet the following three criteria: (1) counseling and ordering of genetic testing was performed by a medical specialist, nurse specialist or nurse, not formally trained as a clinical geneticist or genetic counselor, (2) pre-test counseling was performed, and (3) genetic testing was performed with the primary aim of identifying pathogenic germline variants in patients with cancer.

We considered the study of George et al. [7], published in 2016, to be the first key paper on mainstream genetic testing, and we searched for studies that were published from 2013 onwards, because it is unlikely that earlier mainstream genetic testing initiatives exist. Studies were excluded when predictive genetic testing was performed in healthy individuals. Other exclusion criteria were only a conference abstract being published, being published in another language than English, and lack of availability of a full-text article. Furthermore, we excluded reviews and articles not containing any data (e.g., opinion papers). 

### 2.2. Search Strategy and Databases

Our search consisted of three main criteria: (1) cancer, (2) germline genetic testing, and (3) mainstream genetic testing. The full search was performed in collaboration with a library information specialist, and is shown in Table S1. We searched the following databases: PubMed, Embase, CINAHL and PsychINFO on 4 November 2020. Additional studies were identified through backward and forward reference searching for all included papers. 

### 2.3. Data Collection

All identified studies were imported into Rayyan [14], a web tool for independent screening of abstracts. All abstracts were screened by two authors (KB and MA or KB and MV) for eligibility. 

### 2.4. Outcomes

We assessed the feasibility and quality of care of mainstream genetic testing. Whether the mainstream genetic testing initiatives were feasible for NGHCPs to implement in daily practice was assessed based on two outcomes: (1) the time investment for NGHCPs to discuss and order genetic testing and whether this was acceptable for them, and (2) barriers and facilitators for NGHCPs regarding mainstream genetic testing. 

We assessed quality of genetic care based on the following outcomes: (1) whether some form of training in genetics and genetic counseling was offered; (2) whether an informed consent procedure was described and how informed consent was documented; (3) whether patients carrying a pathogenic variant were invited for post-test counseling at a genetics department; (4) turnaround times for genetic testing (i.e., days between cancer diagnosis, discussing the DNA test, obtaining the blood sample, availability of test result, and disclosure of the test result to the patient); and (5) whether genetic testing was performed according to national guidelines (i.e., whether eligible patients were missed and whether ineligible patients were offered testing unnecessarily).

### 2.5. Critical Appraisal

Two authors (KB and MV) independently evaluated all selected articles using the Quality Improvement Minimum Quality Criteria Set (QI-MQCS), because mainstream genetic testing is considered to improve quality of care [15]. The QI-MQCS covers 16 domains (see Supplementary Methods) for evaluating articles with a quality improvement intervention. These domains mainly focus on the rationale and motivation behind the intervention, how well the intervention can be implemented, whether the intervention is sustainable and has the potential for larger rollouts, and whether the study methods are sufficiently well described. The number of met criteria is noted in Table S2. The QI-MQCS does not supply cut-off values indicating high versus low quality of an article. 

## 3. Results

**Figure 1:**
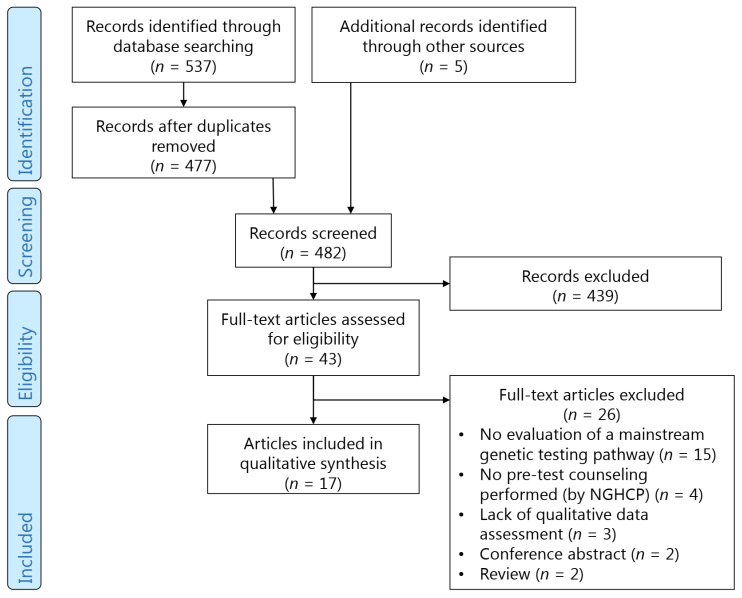
Prisma flow chart.

We identified 537 articles through database searching. Five more articles were identified through reference and citation searching. After removing duplicates, another 439 articles were excluded based on title and abstract. The remaining 43 articles were assessed based on their full text, which led to the elimination of 26 more articles, resulting in 17 articles eligible for our analysis. See Figure 1 for the entire selection process.

### 3.1. Characteristics

We included 17 papers that reported on 15 studies. These 15 studies included nine mainstream genetic testing pathways for patients with ovarian cancer, three for breast cancer, one for breast and ovarian cancer, one for endometrial cancer and one for prostate cancer. Of these 15 studies, 2 were performed in multiple countries and 13 in one country, mainly in the United Kingdom (8 out of 15 studies). All studies, except one [16], were conducted in a research setting. The outcomes on feasibility and quality of care for each study are described in Table S2.

### 3.2. Feasibility

#### 3.2.1. Time Investment for NGHCPs

The time investment for NGHCPs to discuss genetic testing with patients is shown in Table 1. Four out of seven studies described mainstream genetic testing initiatives for patients with ovarian cancer [7,17,18,19,20]. The duration in minutes to discuss genetic testing varied from an average of 8 to 20 min and discussing genetic testing was possible within the available timeframe.

#### 3.2.2. Barriers and Facilitators for NGHCPs

Barriers and facilitators of mainstream genetic testing for NGHCPs [7,8,11,17,18,19,20,21] are shown in Table 2. 

### 3.3. Quality of Care

#### 3.3.1. Training of NGHCPs

In 14 out of 15 studies, a training program was offered to NGHCPs [7,8,9,11,17,18,19,20,21,22,23,24,25,26,27]. One study reported that NGHCPs could attend informational meetings, but they did not receive any specific training in medical genetics [16]. Five studies [8,21,22,26,27] used a training program that was identical to or based on the Mainstreaming Cancer Genetics (MCG) program [7]. This MCG training consisted of online videos. All articles that described the content of the training stated that it covered the informed consent procedure [7,11,17,18,19,22,23,25,27]. Additionally, basic information about the tested genes was provided in multiple training programs. In addition, some training programs provided a detailed explanation of the mainstream genetic testing pathway, health insurance information, and/or billing policies [7,11,22]. In most studies (four out of six) that explicitly described time investment to complete the training, a time investment of approximately one hour was stated [9,11,18,20]. George et al. described a training of less than 30 min [7]. Only Scott et al. described a more extensive training with, among other things, half a day of training by the genetics department about interpreting results and about referral of patients with other cancer syndromes [27]. In four studies, time investment was not explicitly described, but it was stated that the training was based on the training of George et al. [8,21,24,26]. The effect that the training had on, for example, knowledge or skills was only described by Gleeson et al. [18]. Gleeson et al. showed a significant improvement of skills 12 months after the training, but there was no significant difference in knowledge. Skills were self-reported based on the Influences on Patient Safety Behaviors Questionnaire (IPSBQ) [28]. In this questionnaire, NGHCPs assessed whether the training was adequate and offered regularly enough to ensure that all eligible patients were offered genetic testing.

#### 3.3.2. Informed Consent 

All included studies described that patients provided informed consent before NGHCPs ordered a genetic test. In 10 out of the 15 studies, written informed consent was obtained [7,8,9,11,16,18,19,20,24,25,26], and one study described that verbal and/or written informed consent was obtained before testing [23]. Two studies described using the MCG training program, but did not clearly mention if they also used the predefined consent forms developed in this program [21,27]. 

Six studies specified in their article or supplementary material what they considered key topics that should be discussed during pre-test counseling for genetic testing [7,8,17,18,20,26]. All necessary elements for pre-test counseling are shown in Table 3. 

#### 3.3.3. Genetic Counseling for Pathogenic Variants 

In three studies, all patients with a pathogenic variant were invited by the genetics department for post-test counseling by genetic healthcare professionals [7,8,19,20]. In these studies, attendance for post-test counseling was nearly 100%. In the MCG-breast study, two patients out of 117 did not attend post-test counseling at a genetics department [8]. One of these patients contacted the genetics department later. 

In 11 studies, NGHCPs needed to refer patients themselves if the results showed a pathogenic variant [9,11,16,17,21,22,23,24,25,26,27]. In three studies, all or nearly all patients were referred for post-test counseling at a genetics department [9,23,26], although in one of these studies reminder letters for oncologists to refer a patient were necessary [23]. One study reported that 14 out of 18 patients with a pathogenic variant were referred for post-test counseling, and two patients were referred outside of the study period [21]. Attendance for post-test counseling after referral was between 91% and 100% [9,26]. In the ENGAGE study, 76% of patients with a pathogenic variant attended post-test counseling in the European countries, whereas in the US 34% of patients attended post-test counseling [17]. 

#### 3.3.4. Turnaround Times

**Figure 2:**
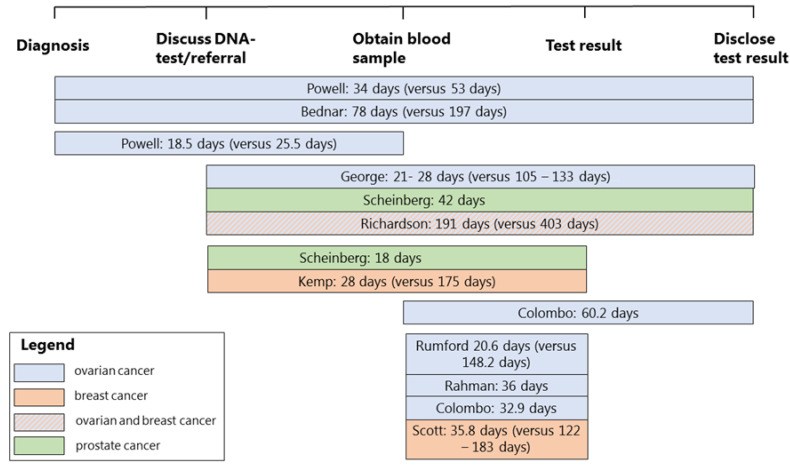
Turnaround times from diagnosis to disclosure of the test result in the patient. Note: if multiple turnaround times were mentioned in one study, these turnaround times are all shown separately. In the articles, turnaround times were reported as calendar days, working days or weeks and these are all shown here as calendar days. Turnaround times of the standard genetic testing pathway are shown between brackets if they were mentioned in the articles.

The turnaround times are summarized in Figure 2. For most studies, a test result was obtained within 3 to 6 weeks after discussing the genetic test with the patient and ordering the test. The longest mean turnaround time was reported by Richardson et al., with 191 days (27 weeks) from discussing the genetic test to disclosing the test result to the patient [25]. An average time was not measured in the study of McLeavy et al., but they reported that 45% of patients in their study received their test results more than 12 months after diagnosis [24]. For all studies that compared turnaround times of the mainstream genetic testing pathway with the standard pathway, there was a reduction in turnaround times during the mainstream pathway [7,8,22,25,26,27]. 

Three studies reported on the time between obtaining the test result with a pathogenic variant and referral to or attendance at a genetics department for post-test counseling [17,21,23]. For most patients, this time varied between 12 working days and 6 weeks [21,23]. In the study of Rahman et al., 2 out of 16 referred patients had a longer time to referral of up to 127 working days. Flaum et al. [23] reported that referred patients received an appointment within 10 weeks at the genetics department. The majority of patients with a pathogenic variant in the ENGAGE study received post-test counseling at the genetics department the same day the test result was available (median 0.0 weeks, range 0.0 to 30.9 weeks) [17]. 

#### 3.3.5. Adherence to Guidelines

Only two studies reported whether genetic tests were offered and/or performed according to current guidelines. In the study performed in Norway, it was assessed for all patients with breast cancer how many patients were eligible for genetic testing according to the Norwegian Breast Cancer Group (NBCG) criteria [16]. Of all patients with breast cancer who did not meet the NBCG criteria, 23% were offered genetic testing by their surgeon or oncologist. Genetic testing was performed in 96% of these patients. Genetic testing was offered to 75% of the patients who did fulfill NBCG criteria, and 96% of these patients got tested.

In the study by Gleeson et al., 93.1% of tested patients with ovarian cancer met national guidelines [18].

#### 3.3.6. Critical Appraisal

The number of QI-MQCS criteria met per article varied between 6 and 13 out of 16, with an average of 8.9. Domains that were described sufficiently in nearly all articles were ‘Organizational motivation’ (16/16 met criteria), ‘Intervention description’ (16/16), ‘Implementation’ (15/16) and ‘Timing’ (15/16). The most insufficiently described domains were ‘Organizational characteristics’ (2/16 met criteria), ‘Penetration/Reach’ (3/16), ‘Adherence’ (4/16) and ‘Health outcomes’ (4/16).

## 4. Discussion

Based on the results of this systematic review, we conclude that it is feasible to incorporate mainstream genetic testing into daily practice because (1) the required time investment was acceptable for NGHCPs, despite the slightly increased workload, and (2) several facilitators and no insurmountable barriers were reported. We conclude that the quality of genetic care was maintained during mainstream genetic testing because (1) these mainstream genetic testing initiatives included genetics training for NGHCPs, (2) a comprehensive informed consent procedure was incorporated to ensure informed decision making, (3) most eligible patients received additional genetic counseling in case of a pathogenic variant, and (4) the turnaround times for genetic testing were comparable or shorter than in the standard genetic testing pathway. The fifth outcome measurement for quality of care, i.e., the proportion of patients receiving mainstream genetic testing that meet the eligibility criteria for testing, is understudied. Based on a combination of the results of our systemic review and those of the systemic review of Scheinberg et al. [12], we postulate that mainstream genetic testing can be successfully implemented in daily practice. 

### 4.1. Feasibility 

#### 4.1.1. Duration and Key Elements of Pre-Test Counseling

It is important that the time investment is reported in articles, because an acceptable time investment is a prerequisite for the implementation of a mainstream genetic testing pathway in daily practice. Compared to standard genetic counseling with an average time investment of 40 to 50 min [29,30], the duration of genetic counseling by NGHCPs, with an average of 8 to 20 min, is shortened substantially. On the other hand, the consultation time with the NGHCP is significantly increased. When mainstream genetic testing becomes part of the standard care, this additional time should be anticipated in the planning.

We identified two key discussion points for pre-test counseling that were described in six studies in this review, i.e., explanation of genes tested and cancer risks, and possible implications of a genetic test for the patients and their relatives [7,8,17,18,20,26]. There was a wide range in number of discussion points that these studies identified as key topics for informed consent, with up to 11 discussion points in the ENGAGE study that they deemed as basic topics of informed consent [17]. The ASCO policy statement describes that it is important to explain the purpose and possible outcomes of genetic tests [31]. Moreover, potential consequences and cancer risks for patients and family members, caused by pathogenic variants in high-, moderate-, and low-penetrant genes, should be discussed, as well as the possibility of finding variants of unknown significance. For example, patients with ovarian cancer need to be informed about the potential impact on their treatment, but also the increased risk of breast cancer for themselves when a pathogenic variant in a *BRCA* gene is identified. Patients should also be informed about the potential increased risk of breast, ovarian or prostate cancer for their family members [32,33].

Shared decision making is essential in genetic pre-test counseling and a lack of time has shown to be an important barrier for shared decision making [34,35,36]. In contrast, research has also shown that reducing the amount of information provided during pre-test counseling is preferred by some patients [37,38]. However, reducing the amount of information could result in less knowledge and more anxiety or distress in patients. Studies measuring anxiety and distress in patients who underwent genetic testing in a mainstream genetic testing pathway have shown low anxiety and distress scores [9,11,20,24,25,39]. To put this into perspective, we need larger studies that compare anxiety and distress scores between the mainstream and the standard genetic testing pathway with pre-test counseling and testing at the genetics department. Ultimately, it is important that the patient can make an informed decision regarding genetic testing, without experiencing an overload of information or unacceptable distress. 

Whether any important information that is needed to make an informed decision is left out by NGHCPs in mainstream genetic testing is not known and should be studied. It is also not known which topics are considered the most important by patients. 

#### 4.1.2. Barriers and Facilitators for Implementation of Mainstream Genetic Testing

The two most important barriers to mainstream genetic testing were inadequate knowledge by NGHCPs and the lack of time during appointments. The latter, however, was only described in one study, by the NGHCPs who had the least experience with mainstream genetic testing [19]. When asked, the extra time investment was acceptable for NGHCPs [7,8,11,17,20]. Lack of knowledge or self-confidence could be solved with a training procedure before NGHCPs discuss genetic testing. 

All these barriers were experienced in a research setting. Gleeson et al. determined which barriers were experienced by NGHCPs that would prevent them from continuing with mainstream genetic testing [18]. These were the lack of local infrastructure or systems to support the mainstream genetic testing pathway, lack of human resources, and lack of funding. It is important that these barriers are considered when implementing mainstream genetic testing in daily practice. Factors that might be insurmountable are lack of human resources and lack of funding, but these differ between hospitals and countries, and the workflow can be adjusted to overcome these factors.

Facilitators for mainstream genetic testing are the offer of a training program, FAQ forms, information sheets, and an approved clinical protocol. In this protocol, it should be stated clearly when NGHCPs can discuss and order genetic tests themselves and which actions are needed to obtain consent, order the genetic test, and when post-test counseling is needed by a genetic healthcare professional. 

### 4.2. Quality of Care

#### 4.2.1. Training

For NGHCPs, it is important to learn about specific key topics that are needed to inform a patient before deciding whether or not to perform the genetic test. Training is therefore an important part of mainstream genetic testing initiatives, as all articles except one described that a training was used for NGHCPs prior to consulting patients [7,8,9,11,17,18,19,20,21,22,23,24,25,26,27]. It was not reported how many of the available NGHCPs actually participated in the studies, and whether some of them declined to participate. Therefore, the results of these studies might be biased, if only the highly motivated NGHCPs participated. It was not described whether these training initiatives were accredited or certified. If the training is accredited, this might motivate more skeptical NGHCPs to participate in and complete the training.

Several modules have been developed to train healthcare professionals in how to provide counseling regarding hereditary diseases [40,41]. Many of these have been developed for NGHCPs and they mainly focus on attitudes about counseling, communication skills and knowledge. Unfortunately, there is a lot of variability among these training modules, and the evidence on their effectiveness is disputable [40]. In most of the studies in our systematic review, the time investment for NGHCPs was 30 min to one hour.

#### 4.2.2. Post-Test Counseling

Although all studies reported that patients should receive post-test counseling by a genetic counselor in the case of a pathogenic variant, the actual referral and attendance rates varied between these studies. The most important difference between these studies seems to be whether or not these appointments for post-test counseling were directly coordinated by the genetics team. In the studies where patients were directly invited by the genetics team, almost all patients attended these appointments [7,8,19,20]. In the other studies, there was a larger variation in the number of referrals and/or attendance of patients for an appointment with a genetic counselor, ranging from 34% to 100% of patients with a pathogenic variant attending post-test counseling [17]. It is striking in the ENGAGE study that the proportion of patients who attended post-test counseling in European countries is substantially higher than in the US. A clear explanation for this difference was not reported, but we can speculate that differences in healthcare costs for patients might play a role. 

It is essential that all patients are offered post-test counseling by a member of the genetics team when a pathogenic variant is identified. Therefore, we recommend that appointments for post-test counseling be directly coordinated by the genetics team. Post-test counseling should be tailored to every patient, and attention should be paid to the practical implications and psychosocial impact of this test result. In addition, there is an important task in guiding patients to inform at-risk family members about cascade testing. Research has shown that even within the genetics departments the uptake of cascade testing is low, ranging from 21 to 44% [42]. Given the time constraints at oncology departments, it is conceivable that this uptake will be even lower without guidance on cascade testing at a genetics department. Post-test counseling is not only important for patients carrying a pathogenic variant, but also for all uncertain variants that are communicated to patients. The clinical implications of an uncertain variant are often limited, but it is important that patients understand these implications. As research has shown that patients as well as NGHCPs often misinterpret the consequences of these variants [17,43,44,45], post-test counseling at a genetics department might be preferable for these uncertain variants as well. 

#### 4.2.3. Turnaround Time

When the genetic test result has consequences for the treatment of cancer, it is important that turnaround times for genetic testing are minimized. For patients with ovarian cancer and prostate cancer for example, there is clear evidence that patients carrying a pathogenic variant in a *BRCA* gene have the highest response rates to PARP inhibitors [5,46]. The longest delay in the turnaround time for the standard genetic testing pathway is probably the time between referral and first appointment with a genetic counselor due to long waiting lists [47]. A prior study showed that the mainstream genetic testing pathway resulted in higher numbers of genetic tests and a lower number of referrals to the genetics departments [48]. Therefore, mainstream genetic testing could be an important facilitator for improving access to genetic healthcare, without increasing the workload for genetic HCPs. This is an additional benefit because the burden on the clinical genetics service is rising [49].

Although most of the included studies reported on turnaround times, it is difficult to compare these results because different time points were used. The most frequently used turnaround time was the time between discussing a genetic test and disclosing the result to the patient, which ranged between 3 and 27 weeks. This illustrates that there are large differences between countries and laboratories. All studies that compared their mainstream genetic testing pathway with the standard pathway showed a reduction in turnaround time during the mainstream genetic testing period. However, it should be noted that most studies compared their turnaround times with periods before the implementation of their mainstream genetic testing pathway, and testing techniques might have been slower in those periods. 

#### 4.2.4. Adherence to Guidelines

Whether patients who received genetic testing fulfilled the criteria as stated in the national guidelines was only reported in two of the included studies [16,18]. As most studies reported on mainstream genetic testing pathways for patients with ovarian cancer, adherence to guidelines is not an important issue because in most countries, all patients with (non-mucinous) epithelial ovarian cancer are eligible for genetic testing [50]. Only one study reported whether genetic testing was performed according to guidelines in patients with breast cancer [16]. In this study, almost 25% of patients who were tested did not meet the eligibility criteria, while 25% of eligible patients did not receive testing. This high proportion of tested patients not fulfilling eligibility criteria is comparable with the 35% of tested patients not meeting the eligibility criteria in the DNA-direct study [51]. In this study, NGHCPs ordered genetic testing for patients with breast cancer without pre-test counseling. The high percentage of ineligible patients in the DNA-direct study illustrates that it is not always clear to NGHCPs which patients are eligible for genetic testing. This should be considered when implementing a mainstream genetic testing pathway for other types of cancer than ovarian cancer. Testing criteria should be simple and clear for NGHCPs to prevent testing of (significant numbers of) patients who do not meet the eligibility criteria. Testing more patients than necessary not only increases healthcare costs, but it can also induce unnecessary stress in patients and produce results that might be challenging to interpret [52]. On the other hand, not testing eligible patients can have serious treatment implications, and it can also result in family members not taking precautions to prevent or detect cancer at an early stage. 

### 4.3. Limitations

This review has some limitations. The results of our review may not easily be generalized to mainstream genetic testing in cancer settings other than ovarian cancer, as the majority (9 out of 15) of these initiatives available in the literature describe a mainstream genetic testing pathway for patients with ovarian cancer. The eligibility criteria for genetic testing in ovarian cancer are much more concise than they are for other types of cancer. Therefore, mainstream genetic testing in other types of cancer is more challenging. 

Another limitation is that there are no predefined and general definitions for feasibility and quality of care for genetic testing. Therefore, other articles might use other definitions to determine these outcomes. 

Lastly, the number of criteria met on the QI-MQCS critical appraisal tool per article was on average 8.9 out of 16. This means that, on average, only 56% of the domains were described sufficiently. The QI-MQCS manual did not always have strict guidance on whether a domain should be scored as ‘met’ or ‘not met’. Therefore, we might have been stricter than necessary, which could have resulted in lower scores. 

### 4.4. Suggestions for the Implementation of Mainstream Genetic Testing

This review shows that there are multiple mainstream genetic testing initiatives which vary in their set-up. There are several elements that should be incorporated into mainstream genetic testing initiatives to increase the likelihood of successful implementation. These recommendations are summarized in Table 4. 

### 4.5. Future Research

Barriers regarding the local infrastructure were mentioned. It is important to explore in detail what these barriers are and to investigate this in different countries, due to differences in healthcare systems. In addition, the proportion of NGHCPs not willing to participate in mainstream genetic testing initiatives and their arguments for this should be studied further, as mainstream genetic testing initiatives can only be successful when a significant proportion of NGHCPs are participating. Mainstream genetic testing may become the new standard of care for specific patient populations, and therefore all NGHCP should be participating in the future. 

Currently, somatic genetic testing is increasingly used simultaneously with or as a prescreen for germline genetic testing [53]. We think that matched germline and somatic testing in a mainstreaming pathway can supplement each other, but further research is needed to evaluate if this is also feasible.

So far, mainstream genetic testing pathways have foremost been evaluated for patients with breast and ovarian cancer. For other types of cancers, there may be different barriers and facilitators that should be evaluated further. 

To determine whether mainstream genetic testing is an acceptable alternative for standard genetic testing, these outcomes should be evaluated more often in comparison to a control group receiving standard genetic testing. 

## 5. Conclusions

The available studies show that mainstream genetic testing for germline variants is feasible in the daily practice of NGHCPs treating patients with cancer. Mainstream genetic testing pathways present an acceptable increase in workload for NGHCPs, and the introduction almost always includes a training procedure. With the introduction of mainstream genetic testing pathways that include training for the NGHCP, the quality of care seems to be maintained. For feasibility as well as for quality of care, it is important that the genetics department has a pivotal role in the mainstream genetic testing pathways, especially in the coordination of post-test counseling of patients with a pathogenic germline variant.

## Figures and Tables

**Table 1:** Time investment for NGHCPs to perform pre-test counseling and disclose genetic test results.

	References
Extra time to discuss genetic test	
No significant added time	[19]
6–10 min (21/64 NGHCPs) and 11–20 min (17/64 NGHCPs)	[18]
8 min	[9,20]
10 min	[11]
20 min	[17]
Extra time to disclose genetic test result	
6–10 min (21/54 NGHCPs) and 4–5 min (8/54 NGHCPs)	[18]
9 min	[11]
NGHCPs (strongly) agreed that discussing genetic testing was possible within the timeframe of a consultation	[7,8,11,17,20]
Workload increased slightly (24/46 NGHCPs) or did not increase (19/46 NGHCPs)	[18]

**Table 2:** Barriers and facilitators of mainstream genetic testing for NGHCPs.

	References
Barriers	
Concerns about added time pressure	[11,19]
Inadequate knowledge about genetics	[11]
Lack of knowledge of VUSs	[11,21]
Lack of local infrastructure	[18]
Lack of human resources	[18]
Lack of funding/unwillingness to allocate funds	[18]
Facilitators	
Supporting materials (training and Frequently Asked Questions)	[7,8,17,20]
Approved clinical protocol	[7,8,17,20]
Information sheets to provide to patients	[7,8,17]
Assistance of a nurse consultant	[11]
Required written test packages	[11]
Education program	[11]

**Table 3:** Necessary elements for pre-test genetic counseling in mainstream genetic testing.

	References
Topics for pre-test genetic counseling	
• Discussing the genes that are tested and their role in the development of cancer	[7,8,17,18,20,26]
• The possible implications of a genetic test for patients (mainly on treatment) and family members	[7,8,17,18,20,26]
• Possible outcomes of a genetic test (i.e., normal result, pathogenic or uncertain variant)	[17,20,26]
• Costs	[17,18,20]
• The possibility of additional pre-test counseling at a genetics department	[17,18]
Informed consent	
• Written informed consent	[7,8,9,11,18,19,20,24,25,26]
• Oral and/or written informed consent	[23]
• Informed consent obtained (not specified as verbal or written)	[17,21,22,27]
Patient information material ^a^	
A summary of the information discussed and/or additional information was provided to the patient in an information sheet after discussing the genetic test	[7,8,9,11,16,17,18,19,20,26,27]

^a^ McLeavy et al. and Rahman et al. used the training developed in the MCG program, but they did not state whether they also used the predefined information sheets developed for patients [21,24].

**Table 4:** Recommendations for implementing a mainstream genetic testing pathway.

Include a training module with
- key topics for pre-test counseling
- an informed consent procedure
Provide clear instructions indicating when patients are eligible for genetic testing
Include FAQ forms and a clear protocol
Invite patients directly for post-test counseling in case a pathogenic variant is found (without the necessity of a referral by the NGHCP)
Close collaboration between genetic and non-genetic departments

## Supplementary Materials

The following supporting information can be downloaded at: https://www.mdpi.com/article/10.3390/cancers14041059/s1, Table S1: Search strategy; Table S2. Outcomes on quality of care and feasibility for each study.
