# Supplementary material for: The Feasibility of Implementing Mainstream Germline Genetic Testing in Routine Cancer Care—A Systematic Review
Source: Cancers (Basel). 2022 Feb 19;14(4):1059. doi: 10.3390/cancers14041059 (PMC8870548; doi:10.3390/cancers14041059)
Supplement: Supplementary file 1 [file cancers-14-01059-s001.zip › Supplementary Methods.pdf]

Supplementary Methods [28]

Quality Improvement Minimum Quality Criteria Set (QI-MQCS) – Version 1.0

ID: ________ Author, year: ___________________________________ Reviewer: _________

Intervention:_______________________________________ Outcome: _______________________________

Domain	Minimum standard	Score
**<u>1. Organizational Motivation:</u> Organizational problem, reason, or motivation for the intervention**<br>♦ Consider quality of care problems; organizational problems; regulations, legal constraints, and external financial incentives at the target organization; or organizational motivation.	Names or describes at least one motivation for the organization's participation in the intervention	Not met<br>Met
**<u>2. Intervention Rationale:</u> Rationale linking the intervention to its expected effects**<br>♦ Consider citations of theories, logic models, or existing empirical evidence that links the intervention to its expected effects.	Names or describes a rationale linking at least one central intervention component to intended effects	Not met<br>Met
**<u>3. Intervention Description:</u> Change in organizational or provider behavior**<br>♦ Consider the presented details that describe the change in the delivery of care, provider behavior, or structure of the organization needed to replicate the evaluated intervention including the involved key personnel.	Describes at least one specific change in detail including the personnel executing the intervention	Not met<br>Met
**<u>4. Organizational Characteristics:</u> Demographics or basic characteristics of the organization**<br>♦ Consider environment (e.g., urban/rural, academic/non-academic), type of care (e.g., primary care), size of the organization, patient mix, staff mix, or reimbursement type.	Reports at least two organizational characteristics	Not met<br>Met
**<u>5. Implementation:</u> Temporary activities used to introduce potentially enduring changes**<br>♦ Consider types of staff involved, activities or methods used such as pilot testing or Plan-Do-Study-Act (PDSA) cycles, staff education, and involvement of stakeholders in introducing the intervention.	Names at least one approach used to introduce the intervention	Not met<br>Met
**<u>6. Study Design:</u> Study design and comparator**<br>♦ Consider the type of evaluation (e.g., post-only, pre-post, time series, parallel control group, randomized groups; same participants assessed multiple times or different samples) / how the authors evaluated whether the intervention worked	Names the study design	Not met<br>Met
**<u>7. Comparator:</u> Information about comparator care processes**<br>♦ Consider details about the control group or the status quo without the intervention (even if there was no formal control group / data), e.g., the existing standard of care / routine care / before the intervention was introduced, or care processes used in the control group.	Describes at least one key care process	Not met<br>Met

**<u>8. Data Source:</u> Data source and outcome definition**<br>♦ Consider the data sources (e.g., routine hospital data, data collected by the study investigator), the data collection method (e.g., survey, interview, objective/subjective measurement) and the outcome of interest is defined (e.g., definition of a reportable patient fall).	Describes the data source and defines the outcome of interest	Not met<br>Met
**<u>9. Timing:</u> Timing of intervention and evaluation**<br>♦ Consider the clarity of the timeline of the intervention, e.g., when introduced, when fully implemented, when evaluated relative to the intervention implementation status, and a clear indication of whether baseline data (defined as before the intervention was introduced) was present.	Describes the timing of the intervention and evaluation to determine the presence of baseline data and the followup period after all intervention components were fully implemented	Not met<br>Met
**<u>10. Adherence / Fidelity:</u> Adherence to the intervention**<br>♦ Consider reporting of compliance with the intervention for the duration of the study, fidelity data on intervention use, or described mechanisms that ensures compliance (e.g., provider reminder integrated in electronic health record that cannot be skipped).	Reports fidelity information for at least one intervention component, or describes evidence of adherence or a mechanism ensuring compliance to the intervention	Not met<br>Met
**<u>11. Health Outcomes:</u> Patient health-related outcomes**<br>♦ Consider patient and non-professional care-giver health-related outcomes (including e.g., quality of life), but exclude satisfaction, provider-behavior (e.g., number of diagnostic tests ordered, knowledge) and process improvements.	Reports data on at least one health-related outcome	Not met<br>Met
**<u>12. Organizational Readiness:</u> Barriers and facilitators to readiness**<br>♦ Consider reported QI resources and culture (e.g., existing QI committee, leadership commitment, prior QI experience, staff attitudes, and education and decision support resources) and results of barriers and facilitator assessments.	Reports at least one organizational-level barrier or facilitator	Not met<br>Met
**<u>13. Penetration / Reach:</u> Penetration / reach of the intervention**<br>♦ Consider the number of units or sites participating in the intervention compared to the available / eligible units (e.g., the number of participating sites without knowing how many sites were initially approached / were eligible is not sufficient).	Describes the proportion of all eligible units who actually participated	Not met<br>Met
**<u>14. Sustainability:</u> Sustainability of the intervention**<br>♦ Consider discussions of sustainability, reference to organizational resources (e.g., costs and necessary commitments) and policy changes needed to sustain the intervention after withdrawal of study personnel and research resources, evidence of enduring changes (e.g. automated electronic reminders), or an extended duration of the intervention period as evidence of sustainability.	Describes the sustainability or the potential for sustainability	Not met<br>Met
**<u>15. Spread:</u> Ability to be spread or replicated**<br>♦ Consider evidence of spread or failure to spread and large rollouts; available resources such as a toolkits, how-to manuals, protocols, or booklets that describe the intervention in detail and could facilitate spread and replication; or discussions of spread potential.	Describes the potential for spread, existing tools for spread, or spread attempts / large-scale rollout	Not met<br>Met
**<u>16. Limitations:</u> Interpretation of the evaluation**<br>♦ Consider whether the interpretation of the reported findings takes the study design (e.g., the lack of comparator) or other evaluation limitations into account; refers to the presented data (not future research / developments or intervention limitations)	Reports at least one limitation of the design / evaluation	Not met<br>Met

Note: QI: Quality improvement. The intervention and the outcome of interest need to be determined before scoring.
