# Supplementary material for: The Feasibility of Implementing Mainstream Germline Genetic Testing in Routine Cancer Care—A Systematic Review
Source: Cancers (Basel). 2022 Feb 19;14(4):1059. doi: 10.3390/cancers14041059 (PMC8870548; doi:10.3390/cancers14041059)
Supplement: Supplementary file 1 [file cancers-14-01059-s001.zip › Table S1.pdf]

Table S1. Search strategy

**Source**	**Search strategy**
PubMed	("neoplasms"[mh] OR "neoplas*"[tiab] OR "paraneoplas*"[tiab] OR "cancer*"[tiab] OR "tumor"[tiab] OR "tumors"[tiab] OR "tumour*"[tiab] OR "malignan*"[tiab] OR "carcinoma*"[tiab] OR "metast*"[tiab] OR "adenocarcin*"[tiab] OR "carcin*"[tiab] OR "HBOC"[tiab] OR "HNPCC"[tiab] OR "lynch*"[tiab] OR "Colorectal Neoplasms, Hereditary Nonpolyposis"[mh]) **AND** ("genetic testing"[mh] OR "genetic test*"[tiab] OR "genetic screen*"[tiab] OR "gene test*"[tiab] OR "DNA test*"[tiab] OR "genetic counseling"[mh] OR "genetic counsel*"[tiab] OR "Genetic Services"[mh] OR "Genes, Neoplasm"[mh] OR "Germ-line Mutation"[mh] OR "germ-line mutation*"[tiab] OR "germline mutation*"[tiab] OR "Genetic Service*"[tiab] OR "germline screen*"[tiab] OR "germline test*"[tiab]) **AND** ("patient centered care"[mh] OR "mainstream*"[tiab] OR "streamline*"[tiab] OR "patient centered care"[tiab] OR "patient focused care"[tiab] OR "focused testing"[tiab] OR "service delivery model*"[tiab] OR "genetic counselor extender*"[tiab] OR "patient navigation*"[tiab] OR "health services accessibility"[mh] OR "health services accessibility"[tiab] OR "oncologist-led*"[tiab] OR "oncologist led*"[tiab] OR "oncologist coordinate*"[tiab] OR "non-clinical geneticist*"[tiab] OR "gynaecologist-led*"[tiab] OR "gynecologist-led*"[tiab] OR "gynaecologist led*"[tiab] OR "gynecologist led*"[tiab] OR "gynaecologist coordinate*"[tiab] OR "gynecologist coordinate*"[tiab] OR "Physician coordinate*"[tiab] OR "Physician led*"[tiab] OR "Physician-led*"[tiab])

Embase	("neoplasm"/exp OR "neoplas*":ti,ab,kw OR "paraneoplas*":ti,ab,kw OR "cancer*":ti,ab,kw OR "tumor":ti,ab,kw OR "tumors":ti,ab,kw OR "tumour*":ti,ab,kw OR "malignan*":ti,ab,kw OR "carcinoma*":ti,ab,kw OR "metast*":ti,ab,kw OR "adenocarcin*":ti,ab,kw OR "carcin*":ti,ab,kw OR "HBOC":ti,ab,kw OR "HNPCC":ti,ab,kw OR "lynch*":ti,ab,kw OR "hereditary nonpolyposis colorectal cancer"/exp) **AND** ("genetic screening"/exp OR "genetic test*":ti,ab,kw OR "genetic screen*":ti,ab,kw OR "gene test*":ti,ab,kw OR "DNA test*":ti,ab,kw OR "genetic counseling"/exp OR "genetic counsel*":ti,ab,kw OR "genetic service"/exp OR "germline mutation"/exp OR "germline mutation*":ti,ab,kw OR "germ-line mutation*":ti,ab,kw OR "genetic service*":ti,ab,kw OR "germline screen*":ti,ab,kw OR "germline test*":ti,ab,kw) **AND** ("patient care"/de OR "mainstream*":ti,ab,kw OR "streamline*":ti,ab,kw OR "patient centered care":ti,ab,kw OR "patient focused care":ti,ab,kw OR "focused testing":ti,ab,kw OR "service delivery model*":ti,ab,kw OR "genetic counselor extender*":ti,ab,kw OR "patient navigation*":ti,ab,kw OR "health care access"/de OR "health services accessibility":ti,ab,kw OR "oncologist-led*":ti,ab,kw OR "oncologist led*":ti,ab,kw OR "oncologist coordinate*":ti,ab,kw OR "non-clinical geneticist*":ti,ab,kw OR "gynaecologist-led*":ti,ab,kw OR "gynecologist-led*":ti,ab,kw OR "gynaecologist led*":ti,ab,kw OR "gynecologist led*":ti,ab,kw OR "gynaecologist coordinate*":ti,ab,kw OR "gynecologist coordinate*":ti,ab,kw OR "physician coordinate*":ti,ab,kw OR "physician led*":ti,ab,kw OR "physician-led*":ti,ab,kw)<br>Filter – Sources: Embase, Embase and MEDLINE<br>Filter – Publication types: Article, Article in Press

CINAHL	((MH "Neoplasms") OR (TI neoplas* OR AB neoplas*) OR (TI paraneoplas* OR AB paraneoplas*) OR (TI cancer* OR AB cancer*) OR (TI tumor or AB tumor) OR (TI tumors or AB tumors) OR (TI tumour* or AB tumour*) OR (TI malignan* OR AB malignan*) OR (TI carcinoma* or AB carcinoma*) OR (TI metast* OR AB metast*) OR (TI adenocarcin* or AB adenocarcin*) OR (TI carcin* or AB carcin*) OR (TI HBOC or AB HBOC) OR (TI HNPCC OR AB HNPCC) OR (TI lynch* or AB lynch*) OR (MH "Colorectal Neoplasms, Hereditary Nonpolyposis")) **AND** ((MH "Genetic Screening" OR MH "Cancer Screening") OR (TI genetic test* OR AB genetic test*) OR (TI genetic screen* OR AB genetic screen*) OR (TI gene test* OR AB gene test*) OR (TI DNA test* OR AB DNA test*) OR (MH "Genetic Counseling") OR (TI genetic counsel* OR AB genetic counsel*) OR (MH "Genes, Neoplasm") OR (TI germ-line mutation* OR AB germ-line mutation) OR (TI germline mutation* OR AB germline mutation*) OR (TI Genetic Service* OR AB Genetic Service*) OR (TI germline screen* OR AB germline screen*) OR (TI germline test* OR AB germline test*)) **AND** ((MH "Patient Care") OR (TI mainstream* OR AB mainstream*) OR (TI streamline* OR AB streamline*) OR (TI patient centered care OR AB patient centered care) OR (TI patient focused care OR AB patient focused care) OR (TI focused testing OR AB focused testing) OR (TI service delivery model* OR AB service delivery model*) OR (TI genetic counselor extender* OR AB genetic counselor extender*) OR (TI patient navigation* OR AB patient navigation*) OR (MH "Health Services Accessibility") OR (TI health services accessibility OR AB health services accessibility) OR (TI oncologist-led* OR AB oncologist-led*) OR (TI oncologist led* OR AB oncologist led*) OR (TI oncologist coordinate* OR AB oncologist coordinate*) OR (TI non-clinical geneticist* OR AB non-clinical geneticist*) OR (TI

	gynaecologist-led* OR AB gynaecologist-led*) OR (TI gynaecologist led* OR AB gynaecologist led*) OR (TI gynecologist-led* OR AB gynecologist-led*) OR (TI gynecologist led* OR AB gynecologist led) OR (TI gynaecologist coordinate* OR AB gynaecologist coordinate*) OR (TI gynecologist coordinate* OR AB gynecologist coordinate*) OR (TI physician coordinate* OR AB physician coordinate*) OR (TI physician led* OR AB physician led*) OR (TI physician-led OR AB physician-led*))<br>Filter – Limit to: Linked Full Text, Abstract Available<br>Filter – Publication type: Journal Article<br>Filter – Source Types: Academic Journals<br>Filter – Language: English
Psycinfo	(exp "neoplasms"/ OR "neoplas*".ab,ti. OR "paraneoplas*".ab,ti. OR "cancer*".ab,ti. OR "tumor".ab,ti. OR "tumors".ab,ti. OR "tumour*".ab,ti. OR "malignan*".ab,ti. OR "carcinoma*".ab,ti. OR "metast*".ab,ti. OR "adenocarcin*".ab,ti. OR "carcin*".ab,ti. OR "HBOC".ab,ti. OR "HNPCC".ab,ti. OR "lynch*".ab,ti. OR "hereditary nonpolyposis".ab,ti.) **AND** (exp "genetic testing"/ OR "genetic test*".ab,ti. OR "genetic screen*".ab,ti. OR "gene test*".ab,ti. OR "DNA test*".ab,ti. OR exp "genetic counseling"/ OR "genetic counsel*".ab,ti. OR "germ-line mutation*".ab,ti. OR "germline mutation*".ab,ti. OR "Genetic Service*".ab,ti. OR "germline screen*".ab,ti. OR "germline test*".ab,ti.) **AND** ("mainstream*".ab,ti. OR "streamline*".ab,ti. OR "patient centered care".ab,ti. OR "patient focused care".ab,ti. OR "focused testing".ab,ti. OR "service delivery model*".ab,ti. OR "genetic counselor extender*".ab,ti.

	OR "patient navigation*".ab,ti. OR "health services accessibility".ab,ti. OR "oncologist-led*".ab,ti. OR "oncologist led*".ab,ti. OR "oncologist coordinate*".ab,ti. OR "non-clinical geneticist*".ab,ti. OR "gynaecologist-led*".ab,ti. OR "gynecologist-led*".ab,ti. OR "gynaecologist led*".ab,ti. OR "gynecologist led*".ab,ti. OR "gynaecologist coordinate*".ab,ti. OR "gynecologist coordinate*".ab,ti. OR "Physician coordinate*".ab,ti. OR "Physician led*".ab,ti. OR "Physician-led*".ab,ti.)
