# Supplementary material for: The Feasibility of Implementing Mainstream Germline Genetic Testing in Routine Cancer Care—A Systematic Review
Source: Cancers (Basel). 2022 Feb 19;14(4):1059. doi: 10.3390/cancers14041059 (PMC8870548; doi:10.3390/cancers14041059)
Supplement: Supplementary file 1 [file cancers-14-01059-s001.zip › Table S2.pdf]

Table S2. Outcomes on quality of care and feasibility for each study

Study, year, country	Study population	Mainstream genetic testing pathway	Training	Feasibility	Quality of care	QI-MQCS[c] met (n)
**George et al., Percival et al., 2016, UK [7, 19]**<br><br>**MCG study**	Patients<br>207 patients with non-mucinous epithelial ovarian cancer.<br><br>NGHCPs<br>13 doctors and 2 clinical nurse specialists.	Pre-test counseling and requesting genetic testing by NGHCP from cancer team.<br><br>Pathway 'v1' (July '13 – May '14): disclosing test result to patient by NGHCP.<br><br>Pathway 'v2' (May '14 – Nov '14): disclosing test result to patient by genetics team (in writing).<br><br>Post-test counseling by genetic counselor for patients carrying a pathogenic variant.	Content: Background of mainstream genetic testing, information about *BRCA* genes, information about the protocol and how to fill in the informed consent form.<br><br>Format: Online videos, freely accessible. Nurses could attend face-to-face training.<br><br>Time investment: Less than 30 minutes.	Time investment:<br>- NGHCPs agreed (on average 4.2/5[a]) that discussing genetic testing was possible within the timeframe of a consultation, but no specific information about the amount of time was provided.<br>- Nurses that performed pre-test counseling in majority of patients reported that no significant extra time was added to a consult.<br><br>Facilitators:<br>- NGHCPs strongly agreed (on average 4.8/5[a]) that supporting materials (training and FAQ) were helpful.<br>- NGHCPs strongly agreed (on average 4.7/5[a]) that it is useful to have an approved clinical protocol.<br>- NGHCPs strongly agreed (on average 4.6/5[a]) that the information sheets to provide to patients were useful.	Turnaround times:<br>- Average time between discussing genetic testing and disclosing result is 3 – 4 weeks in mainstream genetic testing pathway (12 – 15 weeks in standard genetic testing pathway).<br><br>Post-test counseling:<br>- 33/33 patients with a pathogenic variant in a *BRCA* gene attended their appointment at the genetics department.<br><br>Informed consent procedure:<br>- Written informed consent.<br>- Key counseling points described in FAQ form: role of *BRCA* genes in causing cancer, relevance of genetic testing for patients and family members.<br>- Additional information sheets for patients were provided.	George: 10/16<br><br>Percival: 6/16

				Barriers:<br>- 1/5 nurse reported concerns about added time pressures.		
**Bednar et al., 2017, US [22]**	Patients<br>1636 patients with high-grade ovarian cancer.<br>- NGHCPs discussed and ordered genetic testing for 84 patients.<br><br>NGHCPs<br>Gynecologic oncologists or medical oncologists.	Physician-coordinated genetic testing as one of three methods to increase uptake of genetic testing: pre-test counseling, informed consent, requesting genetic testing and disclosing test result by NGHCPs.<br><br>Referral for post-test counseling by genetic counselor for patients with a pathogenic variant.	Content: Informed consent, testing options, billing policies and health insurance coverage, instructions about specimen collection and several examples of possible test results.<br><br>Format: National meetings and conferences and further education by genetic counselors if needed.<br><br>Time investment:<br>Not reported.	Not reported.	Turnaround times:<br>- Time between initial gynecology consultation and completion of genetic counseling: 78 days during mainstream genetic testing period (197 days before mainstream genetic testing pathway was introduced).<br><br>Informed consent procedure:<br>- Informed consent obtained (not specified as verbal or written).<br>- Key counseling points not described.<br>- No additional information sheets for patients were described.	11/16
**Colombo et al., 2018, US, Italy and Spain [17]**<br><br>**ENGAGE study**	Patients<br>710 patients with advanced epithelial ovarian, fallopian tube, or primary peritoneal cancer.<br>- 690 patients received genetic testing.<br><br>NGHCPs	Pre-test counseling, requesting genetic testing and disclosing test result by NGHCPs.<br><br>Post-test counseling by genetic counselor was recommended for patients with a pathogenic variant in a *BRCA* gene.	Content: Background information about *BRCA* genes, genetic testing guidelines and the informed consent procedure.<br><br>Format: Online presentation.	Time investment:<br>- A median of 20 minutes for pre-test counseling was invested by the NGHCP (range 2 – 115 minutes), reported by patients.<br>- 24/32 (strongly) agreed that discussing genetic testing was possible within the timeframe of a consultation.	Turnaround times:<br>- Median time between collecting blood sample and reporting<br>- test result to NGHCP was 4.7 weeks (range: 0.0 – 32.1 weeks).<br>- test result to patient was 8.6 weeks (range: 0.7 – 35.6 weeks).	8/16

	52 oncologists and oncology nurses.		<u>Time investment:</u><br>Not reported.	<u>Facilitators:</u><br>Determined by NGHCPs (after counseling 10 patients):<br>- 27/32 (strongly) agreed that supporting materials were very helpful.<br>- 27/32 (strongly) agreed that it is useful to have an approved clinical protocol.<br>- 27/32 (strongly) agreed that patient information sheets were very useful.	- Median time between disclosing pathogenic variant and appointment at genetics department was 0.0 weeks (0 – 30.9 weeks).<br><br><u>Post-test counseling:</u><br>- Counseling at a genetics department for pathogenic variant: 75.8% of patients in Europe and 35.7% in US.<br><br><u>Informed consent procedure:</u><br>- Informed consent obtained (not specified as verbal or written).<br>- Key counseling points described in PowerPoint presentation: purpose of test and eligibility criteria, general information about genes, possible test results, technical aspects and accuracy of test, economic considerations, possibility of genetic information discrimination, psychosocial aspects, confidentiality, utilization of test result, alternatives to genetic testing, disclosure.<br>- Additional information sheets for patients were provided.

**Rahman et al., 2018, UK [21]**	Patients<br>122 patients with high-grade non-mucinous epithelial ovarian cancer.<br><br>NGHCPs<br>Medical and clinical oncologists.	Pre-test counseling, requesting genetic testing and disclosing test result by NCHCPs.<br><br>Referral for post-test counseling by genetic counselor for patients with a pathogenic variant.	Training is based on training of George et al. [7].	Barriers:<br>- Lack of knowledge of VUS for NGHCPs.	Turnaround times:<br>- Median time between obtaining blood and result was 26 working days (14 – 48 days).<br>- Most patients with a pathogenic variant were referred between 12 – 43 working days.<br><br>Post-test counseling:<br>- 4/18 of patients carrying a *BRCA* pathogenic variant had not yet been referred to a genetics department at the end of the study. Subsequently 2/4 patients were referred after the end of the study (98 and 127 working days after test results).<br><br>Informed consent procedure:<br>- Informed consent obtained (not specified as verbal or written).<br>- Key counseling points not described.<br>- No additional information sheets for patients were described.	8/16
**Kemp et al., 2019, UK [8]**<br><br>**MCG Breast study**	Patients<br>1184 patients with breast cancer.	Pre-test counseling and requesting genetic testing by NGHCPs.	Training is based on training of George et al. [7].	Time investment:<br>- NGHCPs agreed (on average 4.0/5[a]) that discussing genetic testing was possible	Turnaround times:<br>- Time between consultation and test result was on average 4 weeks (in	8/16

	NGHCPs<br>12 oncologists, 8 surgeons and 3 nurse specialists.	Disclosing test result to patient by genetics team (in writing).<br>Post-test counseling by genetic counselor for patients with a pathogenic variant.		within the timeframe of a consultation.<br><br>Facilitators:<br>- NGHCPs strongly agreed (on average 4.7/5[a]) that supporting materials (training and FAQ) were helpful.<br>- NGHCPs agreed (on average 4.0/5[a]) that it is useful to have an approved clinical protocol.<br>- NGHCPs strongly agreed (on average 4.8/5[a]) that information sheets to provide to patients were useful.	standard genetic testing pathway 25 weeks).<br><br>Post-test counseling:<br>- 117/117 patients with a pathogenic variant were offered post-test counseling at a genetics department (115 attended).<br><br>Informed consent procedure:<br>- Written informed consent.<br>- Key counseling points analogous to MCG program.<br>- Additional information sheets for patients were provided.	
**McLeavy et al., 2019, UK [24]**	Patients<br>170 patients with high grade non mucinous ovarian cancer.<br><br>NGHCPs<br>Oncologists.	Pre-test counseling, written informed consent, requesting genetic testing and disclosing test result by NGHCPs.<br><br>Referral for post-test counseling by genetic counselor for patients with a pathogenic variant.	Training is based on training of George et al. [7].	Not reported.	Turnaround times:<br>- 13/29 patients who returned questionnaire received test result >12 months after diagnosis.<br><br>Informed consent procedure:<br>- Written informed consent.<br>- Key counseling points not described.<br>- No additional information sheets for patients were described.	7/16
**Flaum et al., 2020, UK [23]**	Patients<br>480 patients with non-mucinous epithelial	Pre-test counseling, requesting genetic testing	Content: Informed consent procedure.	Not reported.	Turnaround times:<br>- 35/38 patients with a pathogenic variant in a	6/16

	cancer of the ovary, fallopian tube or peritoneum (including carcinosarcoma).<br><br>NGHCPs<br>Oncologists and surgeons.	and disclosing test result by oncologists or surgeons.<br><br>Referral for post-test counseling by a genetic counselor for patients with a pathogenic variant.	Format: Not reported.<br><br>Time investment: Not reported.		*BRCA* gene were referred for post-test counseling at a genetics department within 6 weeks after test result. 3/38 patients were referred between 3 and 5 months after test result.<br>- Referred patients received appointment <10 weeks.<br><br>Post-test counseling:<br>- 38/39 patients with a pathogenic variant in *BRCA* gene were referred for post-test counseling at a genetics department.<br>- Reminder letters to oncologists necessary for 5 patients with *BRCA* pathogenic variant.<br><br>Informed consent procedure:<br>- Verbal and/or written informed consent.<br>- Key counseling points not described.<br>- No additional information sheet for patients were described.	
**Gleeson et al., 2020, Australia [18]**	Patients<br>273 patients with high-grade non-mucinous epithelial ovarian cancer.	Pre-test counseling, requesting genetic testing and disclosing test result by NGHCPs.	Content: Information about mainstream genetic testing (rationale and suggested approach), informed	Time investment:<br>- 21/64 NGHCPs spend on average 6 – 10 minutes obtaining consent for genetic testing and 17/64 spend 11 – 20 minutes.[b]	Adherence to guidelines:<br>- 241/273 of patients met national guidelines.<br><br>Informed consent procedure:<br>- Written informed consent.	13/16

	<u>NGHCPs</u><br>157 medical and gynecological oncologists, advanced medical trainees and oncology nurses.<br>- 47 completed questionnaires.	Post-test counseling by a genetic counselor for patients with a pathogenic variant.	consent procedure and delivering results.<br><br><u>Format:</u> Piloted face-to-face training with a PowerPoint presentation.<br><br><u>Time investment:</u> One hour.<br><br>- NGHCPs scored significantly better (p<0.05) at 'Skills' and 'Environmental context and resources' 12 months after training than pre-training. No difference in knowledge was detected.	- Disclosing test results by NGHCPs took 6 – 10 minutes for 21/54 and 4 – 5 minutes for 8/54.<br>- Workload increased slightly for 24/46 of healthcare providers or had no impact for 19/46.<br><br><u>Barriers:</u><br>Barriers that would prevent mainstream genetic testing from continuing by NGHCPs: 61% identified barriers<br>- Lack of local infrastructure: 31.9%.<br>- Lack of human resources: 27.8%.<br>- Lack of funding / unwillingness to allocate funds: 22.2%.<br>- Lack of influential individuals to lobby for continuation: 15.3%.<br>- Inability to incorporate the service into local health policy and planning: 13.7%.	- Script provided for initiating a conversation about genetic testing including: relevance of genetic test for treatment options, *BRCA* genes and cancer risks, costs, duration of genetic testing, post-test counseling at genetics department if pathogenic variant was found, possibility of additional pre-test counseling at genetics department, genetic test is optional.<br>- Additional information sheets for patients were provided.	
**Grindedal et al., 2020, Norway [16]**	<u>Patients</u><br>131/356 patients with invasive breast cancer were offered genetic testing.<br>- 125/131 received genetic testing.	Pre-test counseling, requesting genetic testing and disclosing test result by NGHCPs.<br><br>Referral for post-test counseling by a genetic	There was no specific training, but only informational meetings were organized in collaboration with the genetics department.	Not reported.	<u>Adherence to guidelines:</u><br>- 47/202 of patients who did not meet NBCG criteria were offered testing.<br>- 69/92 patients who did meet NBCG criteria were offered genetic testing.	9/16

	<u>NGHCPs</u><br>Surgeons and oncologists.	counselor for patients with a pathogenic variant.			<u>Informed consent procedure:</u><br>- Written informed consent.<br>- Key counseling points not described.<br>- Additional information sheets for patients were provided.	
**Powell et al., 2020, USA [20]**	<u>Patients</u><br>43 patients with epithelial ovarian, fallopian tube and peritoneal cancer.<br><br><u>Control group</u><br>101 patients with epithelial ovarian, fallopian tube and peritoneal cancer from other sites.<br><br><u>NGHCPs</u><br>6 gynecologic oncologists.	Pre-test counseling, informed consent, and requesting genetic testing by NGHCPs.<br><br>NGHCPs placed online consult for genetics department to follow up.<br><br>Test results by letter when no pathogenic variant was found and no positive family history.<br><br>Post-test counseling by a genetic counselor for patient with a pathogenic variant.	<u>Content:</u> Not reported.<br><br><u>Format:</u> Training with principal investigator and genetic counselor.<br><br><u>Time investment:</u> one hour.	<u>Time investment:</u><br>- Consenting for genetic testing added on average 8 minutes to the consultation, reported by NGHCPs.<br>- 4/6 NGHCPs (strongly) agreed that discussing genetic testing was possible within the timeframe of a consultation.<br><br><u>Facilitators:</u><br>- 6/6 of NGHCPs strongly agreed that supporting materials (training, information sheet and FAQ) were helpful.<br>- 6/6 of NGHCPs considered that it is useful to have an approved clinical protocol.	<u>Turnaround times:</u><br>- Median time between diagnosis and obtaining blood sample was 18.5 days (25.5 days at other sites with standard genetic testing pathway).<br>- Median time between diagnosis and notification of gene test result was 34 days (53 days at other sites).<br><br><u>Post-test counseling:</u><br>- Referral to genetics department was 100% (genetics department was notified at time of consent).<br><br><u>Informed consent procedure:</u><br>- Written informed consent.<br>- Key counseling points described in checklist: possible results, implications for patients' treatment options, risk of other cancers, possible	12/16

					implications for family members, costs.<br>- Additional information sheets for patients were provided.	
**Richardson et al., 2020, Canada [25]**	Patients<br>165 patients with non-mucinous ovarian cancer, breast cancer <35 years of age, or triple negative breast cancer <65 years.<br>- 49 participated in study.<br><br>Control group<br>537 patients meeting HBOC testing criteria tested through standard genetic testing pathway.<br>- 99 participated in study.<br><br>NGHCPs<br>19 oncologists<br>- 8 completed survey.	Pre-test counseling, requesting genetic testing and disclosing test result by NGHCPs.<br><br>Referral for post-test counseling by a genetic counselor for all patients (regardless of test result).	Content: Information about testing and consenting patients.<br><br>Format: Not reported.<br><br>Time investment: Not reported.	Not reported.	Turnaround times:<br>- Time between referral and return of genetic result was 191 days (sd 174) in mainstream genetic testing pathway compared to 403 days (sd 312) in standard genetic testing pathway.<br><br>Informed consent procedure:<br>- Written informed consent.<br>- Script provided for pre-test counseling (not included in article).<br>- No additional information sheets for patients were described.	11/16
**Rumford et al., 2020, UK [26]**	Patients<br>268 patients with non-mucinous ovarian cancer.<br>- 255 received genetic testing.<br><br>NGHCPs<br>Oncologists.	Pre-test counseling, requesting genetic testing and disclosing test result by NGHCPs.<br><br>Referral for post-test counseling by a genetic counselor for patients with a pathogenic variant.	Training is based on training of George et al. [7].	Not reported.	Turnaround times:<br>- Mean time between obtaining blood sample and returning test result was 20.6 calendar days (range 11 – 42 calendar days). Before implementing mainstream genetic testing pathway,	10/16

					the mean time was 148.2 calendar days.<br><br>Post-test counseling:<br>- Post-test counseling at a genetics department was offered to all patients with a pathogenic variant (31/34 patients attended).<br><br>Informed consent procedure:<br>- Written informed consent.<br>- Key counseling points described in article: discussion of *BRCA* gene, possible implications of the different test results for patient and family members.<br>- Additional information sheet for patients were provided.	
**Ryan et al., 2020, UK [9,10]**	Patients<br>305 patients with endometrial cancer<br>- 300 received genetic testing.<br><br>NGHCPs<br>Gynecological oncology consultants or senior trainees.	IHC and MSI testing on tumor first and targeted *MLH1* methylation if indicated.<br><br>Germline testing only if tumor triage was positive and/or women <50 years or strong personal/family history of Lynch syndrome-associated tumors.	Content: Not reported.<br><br>Format:<br>Individualized training with a clinical geneticist and a genetic counselor. NGHCPs had the opportunity to observe clinical practice in two	Time investment:<br>- Recordings showed that consenting for genetic testing took on average 8 minutes, but this depended on whether a patient was consented before surgery (6 minutes, 29 seconds), on the day of surgery (3 minutes, 58 seconds) or during follow up (10 minutes, 18 seconds).	Post-test counseling:<br>- 13/13 patients who tested positive for Lynch syndrome were offered and received formal genetic counselling at a genetics department.<br><br>Informed consent procedure:<br>- Written informed consent.<br>- Key counseling points not described.	8/16

		Pre-test counseling, requesting tumor and if necessary germline genetic testing and disclosing test result by NGHCPs.<br><br>Referral for post-test counseling by a genetic counselor for patients with a pathogenic variant.	cancer genetic clinics.<br><br><u>Time investment:</u><br>One hour.		- Additional information sheets for patients were provided.	
**Scheinberg et al., 2020, Australia [11]**	<u>Patients</u><br>66 patients with metastatic prostate cancer.<br>- 63 received genetic testing.<br><br><u>NGHCPs</u><br>12 oncologists.	Pre-test counseling, requesting genetic testing and disclosing test result by NGHCPs.<br><br>Referral for post-test counseling by a genetic counselor for patients with pathogenic variant.	<u>Content:</u> Information about genetic testing, counseling and study procedures.<br><br><u>Format:</u> Individual, face-to-face training.<br><br><u>Time investment:</u><br>One hour.	<u>Time investment:</u><br>- NGHCPs spend on average 10 minutes on pre-test counseling (self-reported).<br>- Returning results by NGHCPs took on average 9 minutes (self-reported).<br>- 6/8 agreed or strongly agreed that discussing genetic testing was possible within the timeframe of a consultation.<br><br><u>Barriers:</u><br>- 7/9: time investment for genetic counseling during an appointment was a barrier.<br>- 3/9: inadequate knowledge about genetics was a barrier.<br>- 2/8 did not feel confident they understood VUSs.<br><br><u>Facilitators:</u>	<u>Turnaround times:</u><br>- Median time between pre-test counseling and test results was 18 days (SD 5 days).<br>- Median time between pre-test counseling and disclosing test results to patients was 42 days (SD 30 days).<br><br><u>Informed consent procedure:</u><br>- Written informed consent.<br>- Key counseling points not described.<br>- Additional information sheets for patients were provided.	8/16

				- 8/9: nurse consultant could assist.<br>- 8/9: required written testing packages.<br>- 7/9: education program for oncologist.		
**Scott et al., 2020, UK [27]**	Patients<br>290 patients with breast cancer who fulfilled MCG received genetic testing.<br><br>NGHCPs<br>Two nurses participated.	Pre-test counseling, requesting genetic testing and disclosing test result by NGHCPs.<br><br>Referral for post-test counseling by a genetic counselor for patients with pathogenic variant.	Content: Based on training of George et al. [7], but was complemented with patient observations, a formal consent training and practical training about genetics.<br><br>Format: The practical training included making three generations family history tree. Half a day of training was provided by the clinical genetics service about interpreting test results and referring patients with other cancer syndromes.<br><br>Time investment: A minimum of half a day of training.	Not reported.	Turnaround times:<br>- Average waiting time between genetic testing and test results was 35.8 days. Before implementing the mainstream genetic testing pathway there was a wait time of 12 – 14 weeks from referral to appointment and 4 – 6 months to get test results from time of testing.<br><br>Informed consent procedure:<br>- Informed consent obtained (not specified as verbal or written).<br>- Key counseling points not described.<br>- Additional information sheets for patients were provided.	8/16

[a] scores: 1=Strongly disagree, 2=Disagree, 3=Unsure, 4=Agree, 5=Strongly agree
[b] adjusted from original article after contacting first author, because of an error in the publication

[c] Quality Improvement Minimum Quality Criteria Set
FAQ: frequently asked questions
